# Common Pitfalls in the Interpretation of COVID-19 Data and Statistics

**DOI:** 10.1007/s10272-020-0893-1

**Published:** 2020-06-07

**Authors:** Andreas Backhaus

**Affiliations:** Demographic Change and Aging, Federal Institute for Population Research, Friedrich-Ebert-Allee 4, 65185 Wiesbaden, Germany

## Abstract

Policymakers, experts and the general public heavily rely on the data that are being reported in the context of the coronavirus pandemic. Daily data releases on confirmed COVID-19 cases and deaths provide information on the course of the pandemic.

Policymakers, experts and the general public heavily rely on the data that are being reported in the context of the coronavirus pandemic. Daily data releases on confirmed COVID-19 cases and deaths provide information on the course of the pandemic. The same data are also essential for the estimation of indicators such as the reproduction rate and for the evaluation of policy interventions that seek to slow down the pandemic.

Together with the proliferation of data, however, a number of pitfalls have arisen with regard to the interpretation of the data and the conclusions that can be drawn from them. The aim of this paper is to highlight the most common among these pitfalls given that they have the potential to intentionally or unintentionally mislead the public debate and thereby the course of future policy actions.

The list of pitfalls presented is non-exhaustive. In fact, as the supply of data has increased since the beginning of the pandemic, new pitfalls have emerged in parallel, while others have decreased in relevance; a tendency that seems likely to continue into the future. Beyond explaining some of the current pitfalls, this paper will serve as a more general caveat regarding the interpretation of data in the context of the SARS-CoV-2 pandemic.

## A primer on case fatality rates, infection fatality rates and mortality rates

In the public debate, one can encounter at least three concepts that measure the deadliness of SARS-CoV-2: the case fatality rate (CFR), the infection fatality rate (IFR) and the mortality rate (MR). Unfortunately, these three concepts are sometimes used interchangeably, which creates confusion as they differ from each other by definition.

In its simplest form, the case fatality rate divides the total number of confirmed deaths by COVID-19 by the to-

**Andreas Backhaus**, Federal Institute for Population Research, Wiesbaden, Germany.

tal number of confirmed cases of infections with SARS-CoV-2, neglecting adjustments for future deaths among current cases here. However, the number of confirmed cases is believed to severely underestimate the true number of infections. This is due to the asymptomatic process of the infection in many individuals and the lack of testing capacities. Hence, the CFR presumably reflects rather an upper bound to the true lethality of SARS-CoV-2, as its denominator does not take the undetected infections into account.

The infection fatality rate seeks to represent the lethality more accurately by incorporating the number of undetected infections or at least an estimate thereof into its calculation. Consequently, the IFR divides the total number of confirmed deaths by COVID-19 by the total number of infections with SARS-CoV-2. Due to its larger denominator but identical numerator, the IFR is lower than the CFR. The IFR represents a crucial parameter in epidemiological simulation models, such as that presented by Ferguson et al. (2020), as it determines the number of expected fatalities given the simulated spread of the disease among the population.

The methodological challenge regarding the IFR is, of course, to find a credible estimate of the undetected cases of infection. An early estimate of the IFR was provided on the basis of data collected in the course of the SARS-CoV-2 outbreak on the Diamond Princess cruise ship in February 2020. Mizumoto et al. (2020) estimate that 17.9% (95% confidence interval: 15.5-20.2) of the cases were asymptomatic. Russell et al. (2020), after adjusting for age, estimate that the IFR among the Diamond Princess cases is 1.3% (95% confidence interval: 0.38-3.6) when considering all cases, but 6.4% (95% confidence interval: 2.6–13) when considering only cases of patients that are 70 years and older. The serological studies that are currently being conducted in several countries and localities serve to provide more estimates of the true number of infections with SARS-CoV-2 that have occurred over the past few months.[1]

Finally, the (crude) mortality rate (or death rate) of SARS-CoV-2 is computed by dividing the total number of confirmed deaths by COVID-19 that have occurred in a given location during a certain period of time by the total population present in the same location during the same time period. Therefore, the MR can in principle be computed by dividing a country's COVID-19 death count by its current population. Given that the coronavirus has never infected a country's or location's entire population, the MR will hence be lower than the IFR (and the CFR). However, the computation of the MR is not particularly informative when the pandemic has only been going on for a few months. For example, the global COVID-19 death count has increased more than fivefold between 1 April and 1 May in 2020 (Our World in Data, 2020), rendering any MR computed around 1 April essentially meaningless. Thus, the MR is more appropriately used as a retrospective measure of the damage done in terms of lives lost after a pandemic has run its course.

## Comparability of case fatality rates between countries

In contrast to the IFR, case fatality rates have been available for many countries relatively early on during the pandemic due to their simplicity. Recall that the computation of the CFR only requires the total number of confirmed deaths by COVID-19 and the total number of confirmed cases of infections with SARS-CoV-2. As a consequence, CFRs have frequently been compared between countries. For example, the CFR of Italy has at virtually every point during the coronavirus pandemic exceeded the CFR of South Korea. A naive interpretation of this persistent difference could be that the virus has somehow been deadlier in Italy than in South Korea for unknown reasons. Such an interpretation overlooks that it must be assured first that the CFRs of different countries are comparable. A comparability between CFRs is given only if the confirmed cases that enter the calculation of the CFRs are sufficiently similar in terms of characteristics that are associated with fatalities.

Age is among the most important of such characteristics given the overwhelming evidence that the likelihood of survival is substantially lower for patients at higher ages (Docherty et al., 2020; Dowd et al., 2020). Italy and South Korea are among those countries that have published demographic characteristics of their confirmed cases comparatively early and consistently over the course of the pandemic. Figure 1 compares the confirmed cases by age group in Italy and South Korea. On 19 March 2020, South Korea exhibited a CFR of 1.1%, while Italy's CFR stood at 8.6%. Using data from both countries and from the same date, a simple depiction of the distribution of the confirmed cases across ten-year-age groups reveals that the CFRs of the two countries are not comparable: the cases in Italy are concentrated in the high-age and hence high-risk groups, as 38% of all confirmed Italian cases are at least 70 years old. By contrast, the confirmed cases in South Korea are distributed more evenly across age groups except for a spike in the young age group (20-29). Only 10% of the Korean cases are at least 70 years old. Consequently, the confirmed cases that enter the

1 For a non-exhaustive overview of the serological studies and the associated complications, see e.g. Joseph and Branswell (2020).

Figure 1
**Share of confirmed cases of infections with SARS-CoV-2 by age group in South Korea and Italy**

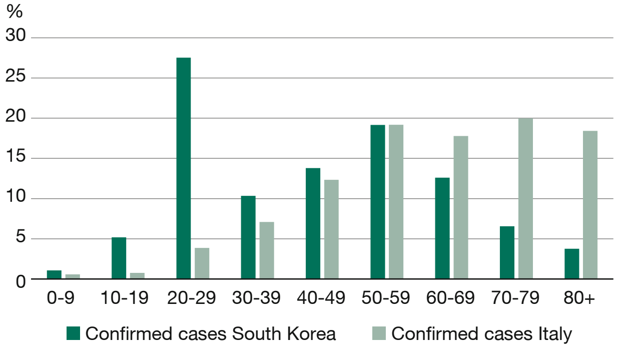

Note: Total confirmed cases on 19 March 2020.

Source: Own depiction based on data from Korea Centers for Disease Control and Prevention and Istituto Superiore di Sanità.

calculation of the Italian CFR are likely to lead to death much more often than in South Korea, resulting in a higher death count and hence a higher CFR for Italy than for South Korea.[2] Dudel et al. (2020) show that changes in the age structure of the confirmed cases over time explain a significant share of the changes in CFRs.

A likely cause for these strikingly different age patterns of the confirmed cases are different testing policies and differences in the timing of testing. South Korea started mass testing relatively early on in the pandemic and many of the early Korean cases could be linked to the 'Shincheonji Church of Jesus' in Daegu. In Italy, mass testing might have started too late to prevent infections from spreading to large parts of the older population at risk. Bayer and Kuhn (2020) further suggest that particularly strong intergenerational ties in Italy could have facilitated the spread from asymptomatic young carriers to the older population.

## COVID-19 death counts and excess mortality

In general, countries use different systems and classifications for recording deaths by COVID-19. These differences may refer, for example, to whether a deceased patient with a severe comorbidity and a confirmed SARS-CoV-2 infection is recorded as having died from COVID-19 or from the comorbidity. Further, countries have changed their standards regarding when a death is counted as a death by COVID-19 over the course of the pandemic. This has led to concerns that countries might either be undercounting or overcounting the deaths by COVID-19.

One way to address these concerns is to look for excess mortality in a given country that is known to have experienced a major outbreak of SARS-CoV-2. Excess mortality can be detected by first collecting data on the total deaths, i.e. the deaths from all causes that are being reported for a given country for 2020, and for previous years. The data from previous years is used to compute the average number of deaths that have occurred in a given country, say Italy, during a given time period, say the month of March. This average is then subtracted from the death count in Italy in March 2020. If COVID-19 led to a significant increase in the death count, the difference between the death count in March 2020 and the average death count of previous years should be positive and somewhat large; it would hence indicate excess mortality due to COVID-19. This difference can then further be compared to the official COVID-19 death count from March 2020. If the difference was larger than the COVID-19 death count, it would suggest an undercounting of COVID-19 deaths, as the reported COVID-19 death count cannot fully account for the observed excess mortality.

The National Statistical Agency of Italy (Istat, 2020) has performed these calculations. They find that until 31 March 2020, deaths in Italy increased by 39% or 25,354 compared to the average of the five previous years. However, only 13,710 deaths have been recorded as COVID-19-related over the same period, which explains only 54% of the observed excess mortality. Hence, if anything, deaths from COVID-19 may have been severely undercounted in Italy despite Italy's already high reported death toll.

## Reporting lags

Reporting lags of the data represent another common pitfall when studying the latest developments of the coronavirus situation. Reporting lags occur, for example, when decentralised offices and institutions do not meet their deadlines for reporting their data to a national agency that then processes and publishes the collected data. Reasons for such non-compliance can be the high workload of local offices during an epidemic or local bottlenecks in testing capacities.

Reporting lags become visible only when updates and revisions to the data are published. Statistics Sweden (2020), the Swedish government agency responsible for producing official statistics, has been very transparent

2 For an early investigation into the demographics of the case fatality rates, see Backhaus (2020).

Figure 2

**Total reported deaths per day in Sweden in March and April 2020**

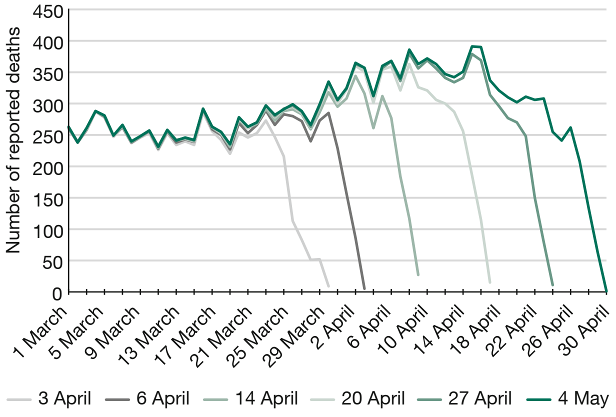

Source: Statistics Sweden (2020), Preliminary statistics on deaths (updated 2020-04-30), Table 8.

regarding the expected reporting lags and the necessary revisions to the reported data on daily deaths in Sweden:

> Statistics on deaths in 2020 refer to data submitted by the Swedish Tax Agency to Statistics Sweden (…) These statistics are updated as new data is made available, as there is a lag in reporting, in particular for the days closest to publication. Statistics from two weeks ago are not expected to change substantially.

Statistics Sweden further provides a vivid depiction of the effects of the various data revisions on the total reported death count per day in Sweden during the months of March and April (see Figure 2): several days before its respective release date, each data series drops abruptly and indicates an unreasonably low death count. Every subsequent data release then substantially revises the death count upwards, with additional but less significant revisions in even later releases. For example, the data release from 6 April reports a total daily death count of 157 for 1 April. However, the data release from the following week revises this initial death count for 1 April upwards by almost 100% to 308. The subsequent releases settle the total death count at 324. Hence, it is important to keep in mind that very recent data are often incomplete and subject to substantial revisions. They are therefore not adequate for immediate use in policy evaluation.

### Sample selection bias

Most often, the data collected and analysed in the context of the coronavirus pandemic do not represent random samples of the underlying population. The same applies to most data being utilised in the social sciences. A consequence of using selected samples is that the insights obtained by means of statistical analysis cannot be trusted to generalise to the overall population.

For example, studies that focus on COVID-19 patients admitted to hospitals or even intensive care perform their analyses on a selected sample, as this subsample of individuals infected with SARS-CoV-2 requiring hospitalisation can be justifiably presumed to differ from the overall population (Williamson et al., 2020).

The issue of generalisability is even more relevant regarding the various serological samples that are being collected and analysed, as they are intended to inform on the true spread of SARS-CoV-2 among the population. Recruitment into these samples often raises concerns about selection: on the one hand, voluntary participation might attract individuals that suspect they may have experienced an infection with SARS-CoV-2 with mild symptoms. On the other hand, analysing samples that were not originally collected for the purpose of testing for antibodies to SARS-CoV-2, such as blood donor samples (Erikstrup et al., 2020), does not resolve all concerns about selection but rather shifts them to a different group, in this case blood donors. The over- or underrepresentation of certain risk groups together with the statistical uncertainty of the rather small serological samples may result in severe misjudgements about the true prevalence of antibodies in the population.

Importantly, sample selection bias is not related to sample size. Hence, increasing the sample size by simply collecting more data will not eliminate the selection problem if the underlying mechanism that governs the selection into the sample is not addressed.

### Endogeneity of policy interventions

It would certainly be worthwhile to evaluate the effectiveness of the various lockdown strategies implemented by governments across the globe in response to the coronavirus pandemic. For that purpose, it might be tempting to rank countries according to the stringency of their respective lockdown strategies and then to simply compare this ranking to a country ranking of the COVID-19 death toll, which would represent the outcome variable that the lockdowns were supposed to affect.

However, such a comparison and equally every regression analysis following the same intuition would suffer from an endogeneity problem. This econometric term is best understood by asking the question: Why have some countries with a high COVID-19 death toll, such as Italy and Spain, chosen a stringent lockdown strategy in the first place? A rather undisputed explanation would be that the situation in these two countries had already been more severe and that the spread of the virus had progressed more than in other countries when a lockdown was first considered. Hence, Spain and Italy had already been heading toward a high COVID-19 death toll when the lockdowns were implemented.

This implies that the allocation of lockdown strategies across countries was not random but driven by early characteristics of the pandemic in the respective countries. These early characteristics would simultaneously determine the stringency of the lockdown and the future death toll. This would result in an underestimation of the lockdown effectiveness, as stringent lockdowns were more likely to be implemented where the situation had already been critical, with dire prospects for the following weeks.

Hence, in the absence of randomly allocated treatment and control groups or countries, as in the case of the coronavirus pandemic, simple comparisons of policy outcomes between groups are potentially highly misleading because other variables might have influenced both the adoption of the various policies and the outcomes.

## Conclusion

From each of the presented pitfalls, a specific lesson can be derived regarding how to handle data in the coronavirus pandemic and what to look out for in the interpretation of COVID-19-related statistics.

First, when utilising different concepts of rates and measurements, for example regarding the lethality, these concepts must be understood, properly defined and appropriately distinguished. Second, when performing comparisons even of the same measure or rate across countries or contexts, one must assure that the underlying data are sufficiently comparable. Third, if there are doubts about the accuracy of the data collected in the specific coronavirus context, other, independently collected data can serve as a tool for validation. Fourth, caution must be applied when interpreting data releases as final or even real-time information because they are frequently revised. Fifth, any interpretation of data and statistics must take into consideration whether selection bias might have affected the collection of the underlying sample. Sixth, when comparing policy outcomes between groups one must be aware of underlying factors that may have determined both the policy choices and the outcomes.
